# Combining experiential knowledge with scholarship in charting the decline of the National Health Service in England

**DOI:** 10.3389/fsoc.2023.1185487

**Published:** 2023-11-21

**Authors:** Graham Scambler

**Affiliations:** University College London (UCL), London, United Kingdom

**Keywords:** auto/biography, NHS, neoliberal politics, funding, auto-biography, biography

## Abstract

The sustained governmental assault on the National Health Service (NHS) in England during post-1970s financialised or rentier capitalism has received considerable attention by the research community. There is some evidence, however, that many of those members of the public who have not had occasion to use the NHS remain largely ill-informed about the extent of, and reasons for, its present troubles. In this paper I offer an auto/biographic account of my own recent experiences as a patient with type 2 diabetes and subsequent polymyalgia in both primary and secondary care. I then deploy analytic induction to consider, and explain, my personal travails against the background of the shifting nature of doctor-patient interaction occasioned by governmental politics in relation to the NHS. The result is an illustrated story of the decline of health care at a political juncture when the ever-expanding capital assets of a tiny minority of the population trumps the health care needs of the population as a whole. The present impoverishment of management and care must be understood with reference to wider aspects of macro-social change. The paper concludes with some ideas about how to (re)fund a severely ailing NHS.

## Introduction

This contribution seeks to combine a research-based account of changes in the English National Health Service (NHS) with my recent and ongoing personal experience of long-term illness and resultant contact with local medical practitioners in primary and secondary care. In the first section the scene is set via an abbreviated account of the quite rapid evolution of health care policy in England. The central theme here is the privilege being accorded to the concept of the market and to what might be termed the incremental privatisation or “Americanisation” of health care facilities and services. In the second section I introduce and address my own experiences and the methods adopted in this paper, drawing on a mix of auto/biography and analytic induction. I attempt to both illustrate the changes to the NHS as I experienced or “came up against” them, and to suggest that my series of encounters allows for a degree of extrapolation to, and a two-way or dialectical relation to, the national picture. In the third section I reflect on the social and sociological ramifications of this narrative. In the fourth and final section the analysis is more explicitly sociological. I advance research-based explanations for the policy changes detailed in section one and define these changes as regressive and rooted in policy-based evidence rather than evidence-based policy. I then go on to establish the explanatory salience of macro-social transformations linked to post-1970s financialised or rentier capitalism.

### The evolution of policy: the NHS under threat

As I write this the English National Health Service (NHS) is beset by any number of emergency incidents, which is reflective of a long-term decline in its funding which the advent of the COVID-19 pandemic served to expose. In this contribution to understanding and explaining what is now widely perceived to be a crisis in both the health and social care systems, a crisis with long tap roots, I combine an account based on scholarly research with what I classify as “experiential knowledge”. The latter draws on my personal and ongoing experience of primary and secondary care following the onset of polymyalgia in March of 2022, compounding the continuing accommodation and management of type 2 diabetes. By way of context, it should be noted that plans to radically “reform” the NHS were planted and began to germinate during Thatcher’s period of office in the 1980s, for all that they were largely arrested by alert and antagonistic public opinion: the strength of the public’s commitment to the NHS had yet to be effectively undermined (Pollock and Leys, 2004). The introduction of a makeshift “internal market” via the NHS and Community Care Act of 1990, which encouraged private providers, was as far as she could go. This half-way house or pseudo-market sat somewhere on a spectrum between a bureaucratic command and control economy and a private free market, but it was a sign of things to come. Of the reforms introduced by Thatcher’s successor, John Major, the introduction of Private Finance Initiatives (PFIs), which were enthusiastically endorsed by the Blair/Brown New Labour regimes between 1997 and 2010, is of special salience. PFIs allowed for the private sector to build, and own, new hospitals and other health care facilities, which they then leased back to the NHS, often at higher rates than would have been the case with government funding and on the basis of 20–30 year deals. They appealed to successive governments because PFI building and refurbishment did not appear on governments’ books: they represented an investment of private not public monies. Predictably enough, PFIs subsequently became major contributors to the indebtedness of many NHS Trusts. Thomas (2019) notes that PFIs have become a postcode lottery, and he estimated that 20 years after they were introduced only around £25 billion of the £80 billion expected total cost had been paid; that is, less than a third of the final price, with £55 billion still to pay. This indebtedness was exacerbated by the cuts and austerity measures following the financial crash of 2008–9.

If the future of the NHS as a universalistic and inclusive health care system was rarely openly challenged prior to 2010, a lot was happening behind the scenes. There is evidence of protracted and persistent private sector lobbying prior to the 2010 election (Leys and Player, 2011). Despite David Cameron’s pre-election promise that there would be no top-down reorganisation of the NHS by a Conservative government, Health Minister Andrew Lansley, well primed by for-profit health care providers, was in fact already well advanced in his preparations for what was to become the Health and Social Care Act of 2012. This complex and far-reaching piece of legislation further opened the door to for-profit providers of health care. It should be noted in this connection that firm evidence already existed that privatised health care: (i) augments costs because it requires an expanded bureaucracy that comes with contracts, billing and litigation; (ii) encourages “cherry-picking”, with the private sector focusing on the most lucrative work, like hip and knee replacements; (iii) opens the way for fees to be introduced as services are cut and hospitals pushed into – often PFI-induced – debt, with for-profit companies “coming to the rescue”: (iv) prioritises cost of care over quality of care; (v) leads to rationing, another trigger for patients to “go private”; (vi) under cover of commercial confidentiality makes it impossible to properly scrutinise public spending via contracts with private providers that are primarily oriented to their shareholders; and (vii) promotes a fragmentation of health care services as these are refashioned according to market principles (see Scambler, 2019).

But if the Health and Social Care Act opened the door for the promotion of for-profit health care, a tranche of further intricate, extra-legal “devices” were put in place by stealth and under the public radar. Lansley’s successor as Health Minister, Jeremy Hunt, who was already on record as personally favouring NHS privatisation (see Stone, 2016), championed a series of initiatives which, whatever merits might be claimed for them, were also designed to accelerate the privatisation of health care in England. For example, a new model of care via Accountable Care Organisations (ACOs) was introduced, and on Hunt’s watch a plan devised to “bundle up” services into “giant contracts” awarded by Clinical Commissioning Groups (CCGs) – and local authorities – to ACOs. ACOs comprised Multi-Speciality Community Providers (MCPs) and Primary Acute Services (PASs), which could involve private and/or public providers. ACOs could subcontract and sub-subcontract for services. And MCP and PAS providers could form Special Service Vehicles, a device to clandestinely engage the likes of private health insurers, property companies and investment bankers. A local service operating under the NHS brand could subsequently be owned by an American private equity company. Following up on this last point, a good deal of attention had been paid recently to the involvement of Centene/Operose in health care in England.

Operose was formed early in 2020 when the American company Centene Corporation brought together its UK subsidiaries, The Practice Group (TPG) and Simply Health. TPG had been acquired by Centene in 2016. In January 2020 Centene increased its stake in UK-based health care by investing in Circle Care (a 40% stake according to Company House). In February 2021 Operose acquired AT Medics and its considerable number of GP surgery contracts in London. Previously owned by six GPs directors, AT Medics had been operating 49 practices across 19 London boroughs, providing services to around 370,000 people, with 900 employees. On being acquired by Operose, its directors resigned and were replaced by Samantha Jones (CEO of Operose, ex-head of NHS England’s new care models programme, previously chief executive of Epsom and St Hellier University Hospitals and West Hertfordshire Hospital Trusts, and later PM Boris Johnson’s health adviser). A case brought by a patient at an AT Medics surgery protesting the award of dozens of contracts to Operose was dismissed by a High Court Judge in February 2022.

Two observations are in order at this point. The first is that it was under the rubrics of “modernisation”, “partnership working”, and “patient/consumer choice” sponsored by Blair and Brown’s New Labour governments that initiatives like the formation of AT Medics were positively welcomed and encouraged. AT Medics was set up in 2004. The six founding “doctorpreneurs” won several contracts under conditions allowing GP companies to run publicly funded GP surgeries and to employ doctors; patients did not pay fees but ‘GP consortia’ companies could profit from public NHS funds to run GP surgeries. So it was New Labour who pioneered new business models that the Conservatives went on to develop post-2010 and to refine post-2020. At present (June 2022) the Operose website lists contracts for 20 GP surgeries, plus one treatment centre in Birmingham (plus nine ophthalmology services). This website also now lists the contract for AT Medics to provide services for all of Croydon and some of the South-West London Clinical Assessments Service. With the addition of the AT Medics contracts, the company will have 69 GP surgeries and become the largest GP surgery network in the UK. Second, a well-advertised BBC Panorama programme shown on 13 June 2022 drew on the research of an undercover reporter, who found that Operose employs less qualified US-style Physician Assistants (PAs) to see patients without adequate supervision. Reports from administrative staff confirmed that some correspondence had not been processed and seen by a GP or pharmacist for up to six months. The undercover journalist working as a receptionist at one of the company’s London surgeries quoted a GP as saying that they were short of eight doctors and that the practice manager said they hired less qualified PAs because they were “cheaper” than GPs. This new model represents as abandonment of the principles on which the NHS was founded. No longer is the NHS insulated from the profit motive. Centene/Operose are in it to make money and they and their like are permeating England’s health care with the blessing of a succession of governments (Mann, 2022).

The most recent top-down reorganisation of the NHS came via the Health and Social Care Act of 2022. This Act established Integrated Care Systems (ICSs) as commissioners of local NHS services, whilst also granting the Minister ultimate authority over the health service. Specifically, the two component parts of the ICS – the Integrated Care Board (ICB) and the Integrated Care Partnership (ICP) – are to have statutory status and will collectively hold the ICS’s legal powers and responsibilities. ICBs will be responsible for the NHS functions of the ICSs, while the ICPs will oversee their wider public and population health work. What this means in effect is that Clinical Commissioning Groups will be absorbed into their local ICSs; and their commissioning powers and most of their staff will become part of the ICS body. The British Medical Association had expressed concerns at these projected changes at the Bill stage, seeking assurances that there would be: appropriate clinical and patient involvement at every level of ICSs; a default option for establishing the NHS as provider of NHS contracts to protect the NHS from costly procurement and fragmentation of services; guarantees that private providers would not exercise undue influence by sitting as members of NHS decision-making bodies; and safeguards and limitations over the Minister’s powers to avoid unnecessary political influence in NHS decision-making. It is already hard not to see this intervention as too little too late, but then the BMJ was primarily focused on its members’ interests.

Other critics have been more forthright. One such critique runs as follows: now the old system of Clinical Commissioning Groups has been replaced by ICBs, it is up to NHS England, not parliament, to decide who each ICB will be responsible to. It could be, for example, that ICBs might be able to challenge allocations and thereby, in effect, to select patients. New groups of people could be excluded from NHS care, as certain migrant people currently are. Another compelling criticism is that after many years of NHS under-funding, and then COVID, the inevitable result will be more rationing and care will become a postcode lottery. It will, it was presciently claimed, become harder to see a GP and the NHS could well become a kitemark for providers. For-profit companies will receive taxpayer money to deliver procedures, and shareholders will be prioritised over reinvestment in the NHS. These may be early days, but there is no doubt that a door deliberately left ajar in 2012 to welcome and incentivise private providers has been opened further by the 2022 legislation (see Scambler, n.d.). These are issues I return to in the final section of this paper, but it is now time to address my personal experiences.

### Knowledge via experience: methods and the unfolding of events

Auto/Biographic data can put compelling, including emotional, flesh on otherwise bald skeletal accounts of social phenomena by appending experiential to scholastic knowledge (see Twinley and Letherby, 2022). It offers a return route from the personal to social structural and cultural relations (Ellis et al., 2011). In their discussion of auto/biographical approaches to researching death and bereavement, Brennan and Letherby (2017) extend this argument by contending that auto/biographical studies can constitute a challenge to “traditional” claims to objectivity both by recognising and factoring in the personhood of researchers and respondents and by providing a more intimate access to and way of analysing the complexity of the relations between self and other. My personal narrative in this contribution contains a fair amount of detail and covers a period of nearly a year between March of 2022 and February 2023. I had for a number of years been experiencing type 2 diabetes, for which I was taking routine medication when, out of the blue, I began to experience severe pain in my joints. The discomfort was omnipresent, accompanying all movements, and was particularly inhibiting when lifting or carrying. It was even painful to change position in bed. Adopting a not uncommon – and characteristically British – policy of “wait and see”, I tolerated this for some weeks before seeking help. As “encouraged” by my local GP surgery, I went online to request a face-to-face appointment, incorporating a suggestion that I might be suffering from polymyalgia (which had afflicted my mother and which had also been confidentially raised as a possibility by a professorial clinical colleague via DM on Twitter). I was granted a phone call, a mode of contact that I was instinctively reluctant to regard as “a consultation”. Despite being registered with the practice for nearly 20 years, “GP A” who rang me was not known to me. On being told that I had experienced a bout of COVID two months previously, she diagnosed long COVID and was reluctant to consider alternative diagnoses. She prescribed strong painkillers to be taken for six to eight weeks. These painkillers had absolutely no effect and I stopped taking them after a month. At this point an insistent phone call to the practice secured a face-to-face GP appointment, once again with a hitherto unknown practitioner, “GP B”. “GP B” was very receptive and helpful, made a provisional diagnosis of polymyalgia and arranged for both a blood test and a consultation with the local consultant rheumatologist. In the meantime, he started me on a low dose of steroids and, at my prompting, we discussed the likely disruptive effect of the steroids on my glucose levels. So around two months after the onset of symptoms, and a month after my initial contact with the surgery, some progress was being made.

I was not to see “GP B” again, though he did later phone me with the blood test results to confirm that the diagnosis of polymyalgia was the front runner and to see if I had yet seen the consultant rheumatologist. I replied that I had not at that point but that an appointment had been arranged. I was never to hear from him again. When I saw consultant rheumatologist, “Consultant 1”, at my “local” – it was a half-hour drive – hospital, she advised upping the dose of steroids and prescribed a selection of other drugs to mitigate any negative effects of the steroids. She asked me what I had done prior to retirement and, on discovering that I had been Professor of Medical Sociology at UCL Medical School, initiated a relaxed conversation about shared experiences. She gave me a personal mobile number and told me to let her know if any problems arose. This consultation led to a series of blood tests and follow-up encounters at the same hospital, which was her base. I was also referred to have a scan – incidentally from a privatised facility – to check that I was not suffering from temporal arteritis, a potentially serious condition that can lead to irreversible sight loss (I wasn’t). Over time, the steroids alleviated the symptoms of pain and discomfort without eliminating them. The principal residual pain was, and has remained up to the present, in the fingers and hands. But a lot of water has passed under the bridge between then and now.

Somewhat to my surprise there was no attempt on the part of my general practice to either monitor the effect of the steroids on my glucose levels, which did indeed become predictably unpredictable, or to assume general responsibility for my continuing treatment and care. In fact I had to return for a second visit to have the scan to check for temporal arteritis because my unmonitored glucose level was too high on the first visit to proceed. I wrote to the senior GP partner at this point articulating my concerns in what I hoped was a sensitive fashion, but she did not reply. I subsequently redacted all personal and local details and made this letter available as an ‘open letter’, the final two paragraphs reading as follows:

‘I am sure this is all stressful for everyone working in the NHS, the more so if they are aware of the politics. It is of course stressful for their patients too. Patients, as you will know only too well, are now angry when they cannot see ‘their’ GP, or indeed any GP. Receptionists too are in the front line. What I would say is that we patients are right to be angry at the effective termination of continuity of primary care and at what is without question a deterioration in the service, with worse to come. The problem is that few patients either realise the constraints within which you work or that this is all a predictable consequence of the political strategy deployed by central government.

I would like to see blame apportioned appropriately. In particular I would personally like to see pithy posters in every NHS surgery and clinic explaining that staff are doing all they can to meet growing patient demand, but that services have been impacted by central government underfunding, which has long affected staffing levels, hospital beds etc., but which has been made worse by restraints on doctors and nurses trained overseas, COVID, practitioner burn out, exhaustion, and so on. Might something like this be desirable/possible? I think health workers and patients should be united in standing up for the NHS.’

It was in fact only courtesy of “Consultant 1” that the diabetes issue was addressed. She referred me across to “Consultant 2”, who duly fortified my existing diabetes medication with another top-up drug. Over the following months I had repeated blood tests and saw “Consultant 1” quite regularly, after a while at a more convenient out-patients clinic, and I was at the same time monitored also by “Consultant 2”. Whereas initially my now-multiple repeat prescriptions were available from my local community pharmacy, this was discontinued due, I was informed, to a shortage of delivery drivers. From that point on I switched to another community pharmacy and attempted to arrange for the relevant prescriptions to be requested in timely fashion from my general practice: these would normally take two or three working days to be processed and made ready for my collection.

I negotiated another face-to-face appointment, this time with “GP 3”, yet another stranger to me, to ensure that all the drugs I required were recorded in my file. He told me that he was not “allowed” to add any drugs to the list and that I would have to see the practice pharmacist. “But your’e a doctor! Are you telling me that you cannot check and update my file yourself?” “Sorry.” An appointment with the pharmacist was not possible for two or three weeks. When I did see her, I went through each of the nine drugs I was taking at that point. During this period I became increasingly dependent on “Consultant 1” to compensate for the tardiness of the general practice: I would text her, with profuse apologies, to ask her to send through prescriptions for whatever drug I was running out of to the local community pharmacy for me to collect. This unhappy routine was eventually to reach a climax when I was thwarted in acquiring more steroids (which as the card in my wallet affirms, it is not advisable to run out of). I visited the practice personally on four occasions over two or three days to sort out any apparent confusion. On each occasion the receptionists were concerned and helpful, and on each occasion the prescriptions were in the event not signed off by the duty GP, this despite me showing the receptionists my copies of letters from “Consultant 1” to the practice that explicitly confirmed that she had prescribed the relevant drugs and new copies of these letters being taken (they should of course have been in my practice file anyway). In the end, in despair, I filed a complaint online, this time making my personal experience and knowledge of healthcare and of the pressures GPs and other health workers were under clear. Within hours I was rung by the practice manager, offered an apology, and reassured that it would not happen again. I expressed my scepticism.

### Knowledge via experience: reflections

Familiarity with the research literature on long-term illness is of limited help in coming to terms with its day-to-day intrusion. In my favour was the fact that I was retired and therefore not obligated to fulfil routine work-related tasks. Coming to terms with type 2 diabetes, a common enough issue, had not troubled me. I was aware that severe and unrelenting weight loss through dieting could remove it, but I was no less aware that people who take this option tend to relapse sooner rather than later. Polymyalgia occasioned different problems. These fell into several categories. First, it was and remains painful, even when mitigated by strong medications. Give the concentration of this pain in the fingers, hands and wrists, the left marginally more than the right, the price paid in loss in functional engagement has been considerable: my hands and arms have lost their powers of leverage. This is an impairment that exacts a continuing cost. Second, there is continuing uncertainty about both the prognosis, though polymyalgia might typically be expected to loosen then relinquish its grip between 18 and 24 months, and the likely medium-to-long-term effectiveness of quasi-experimental cocktails of drugs. Third, and perhaps of most salience, there is a lingering uncertainty about the precision or “completeness” of the diagnosis of polymyalgia. “Consultant 1” has been refreshingly open-minded here. Is it possible that long COVID triggered, or is even mimicking, polymyalgia? And is it possible that the unremitting pain in the fingers is mostly or partly a symptom of rheumatoid arthritis? Furthermore, how might the undoubted iatrogenic effects of the drug regimens be factored in? It is something of a cliché in medical sociology that patients abhor uncertainty, sometimes even preferring, at least in the short term, negative surety over continuing doubt. I confess to seeking answers to constrain and retract uncertainty’s boundaries; but I am only too aware that experts like “Consultant 1” are necessarily extemporising, especially given the lack of data around COVID in general and long COVID in particular. In my case, as I awake in the morning and ritualistically clench my fists and flex my fingers to appraise the day’s likely level of discomfort, I am confronted not only by “rational clinical uncertainty” but by other potentially intrusive causal factors like the typical impediments of ageing and the relatively unstructured, even anomic, day that lies ahead. Just what is doing what to me, and how will it pan out over what time scale?

What of my views of my encounters with the medical profession? I have been very impressed by “Consultants 1 and 2”, though I may well have had a degree of preferential access, especially to “Consultant 1”, by virtue of my professorial past in UCL Medical School. This was not a privilege I sought by mentioning/trading on my university position – I was asked in passing about my career by “Consultant 1” – but I suspect I was subsequently and remain a beneficiary. But between them, “Consultants 1 and 2” have simultaneously applied their specialist expertise *and* substituted for the lack of engagement or continuity in GP care. I would put it more strongly: my experience of GP care throughout this lengthy episode has been close to abysmal. Like many others I have found it exceptionally difficult to arrange face-to-face consultations, this despite being personally informed by one GP – in the midst of my period of illness – that the practice partners had met and decided that face-to-face consultations were to be the default option. Every time I attended the local surgery, usually for simple procedures like blood tests, the waiting room was virtually empty, even in peak mid-morning shifts. When I asked where the twelve GPs associated with the practice were, I was told they were either in their rooms, at one of the other two surgeries covered by the practice or working from home.

These observations, or impressions, coalesce into a series of propositions that I will seek to contextualise and examine in more depth and more sociologically in the concluding section of this paper.

1. From my experience, which routine reports on the mainstream and social media confirm are not unusual, GP care has suffered a marked deterioration. The differences between the GP care available when I was a child in the 1950s and now are almost beyond characterisation. In the 1950s I could turn up and queue for an appointment with my GP and get one in either daily morning or evening surgeries. If I needed a home visit, I could have one on the same day. It will be objected that 70 years on we now inhabit a more complex, highly differentiated world; rapid population growth has taken place, especially of third and fourth agers; concepts of illness and disease have expanded; medical interventions and technologies have become more sophisticated; and the provision of clinical treatment and care has become much more expensive in real terms. All this is true. But we should note and not downplay the vivid contrast between GP access during my childhood and GP access now.
2. The degree and rate of deterioration in GP services has accelerated since the Thatcherite 1980s. The notion of ‘deterioration’ is pivotal here. Back in the 1980s I was asked by a group of consultant neurologists to specify the criteria for good quality care in relation to epilepsy. When I did so, one consultant responded by politely protesting: “That’s all very well Graham, but we simply do not have time to do this.” My response is germane: “Then do not claim to offer good quality care. The best you can do in unfavourable circumstances does not necessarily equate to good quality care.”
3. It is important that GPs acknowledge that the service they currently offer *has in fact deteriorated*.
4. It does not follow from this deterioration in primary care that GPs and their colleagues in allied professions are culpable. They might be on occasion, but they are more often not.
5. The oft-heralded rapid displacement of face-to-face consultations by phone calls is not *primarily* a rational innovation but rather a GP practice coping device. This is not to deny any future role for phone calls, emails, telemedicine and so on (far from it); but it is to insist that the current shift in modes of contact is GP-led not patient-led. I have found phone calls with “Consultant 1” and “Consultant 2” convenient and helpful, but with GPs nominally responsible for continuing care much less so. The point to emphasise is that it is simply disingenuous to suggest that this switch from actual to virtual consultations is the result of patient choice. It is not! It’s a mechanism that helps GPs and their colleagues get through their day-to-day workloads.
6. What can easily go missing in virtual dialogue are those aspects of human relations that find their expression in gestures like nods, smiles and “bodily concern”. As a prospective patient I have happily had very little contact with doctors over the years, but perhaps it is not unsurprising that now, in my mid-70s, and drawing on what little experience I have had as a patient, I want, I almost wrote “demand”, to sit down and speak to someone face-to-face who manifestly cares about my pain and discomfort and the stresses they occasion. I have had this with “Consultants 1 and 2”, but it has been missing from general practice.
7. I have argued that GPs might put up posters in their surgeries apologising for *what now really is a poor service* and stating that the medical and allied staff are doing the best they can in new and challenging circumstances that have their origins in the underfunding of both primary and secondary care plus the sequelae of COVID etc. Patients are (often) right to be angry when they cannot see their GPs, but they are (equally often) wrong when they take this anger out on GPs and their colleagues.

### Why this impasse? How might sociology help explain it?

In this section an attempt is made to place both the moves of successive governments to ‘reform’ the NHS and to promote private health care and my own experiences seeking help with long-term illness in context. Here scholarship informs experience. What the data show is that the top-down reorganisations of the NHS have been accompanied by constraints placed on funding, most conspicuously during the decade of austerity introduced by the Cameron government from 2010. Drawing on resources from the King’s Fund (2022) and the British Medical Association (BMA) (2022a,b,c), a summary of pertinent statistics on annual expenditures on the NHS in England and on changes affecting primary and secondary care reads as follows:

- Funding for health services in England comes from the Department for Health and Social Care’s budget. Planned spending for 2022/23 is £180.2 billion, the majority of which will go to NHS England (£152.6 billion), with the remainder allocated to other national bodies for spending on other health-related functions such as public health. After several years showing modest increases, the Department’s spending in 2020/21 and 2021/22 included funding to respond to COVID, with the result that the Department’s budget grew rapidly between 2019/20 and 2021/22 before falling in 2022/23. It is projected to increase by 1.2% in real terms over the next two years. To provide further context it should be noted that the NHS continues to face severe financial pressures, with Trusts across the country spending more than they are bringing in. NHS England in 2013 said it faced a funding gap of £30 billion by the end of the decade. Despite this, the NHS was asked to find £22 billion in savings by 2020. The Nuffield Trust and King’s Fund have shown that tight spending and increasing demand for services have already led to some treatments being rationed and the quality of care in some areas being diluted.
- Referring to the hospital sector, COVID laid bare the fact that England does not have enough critical care beds. Bed shortages alongside high occupancy are unsafe for patients and staff. Data for the second quarter of 2022/23 indicate that bed occupancy levels in England have risen substantially and have passed the recommended Sage threshold again. In fact, since 2010 average bed occupancy has consistently surpassed 85%, the point at which safety and efficiency are at risk. Coming into the pandemic, England had an average bed occupancy of 90.2% in 2019/20, though local variation in supply and demand have seen many Trusts regularly exceeding 95% capacity in the winter months. Prior to COVID, the total English NHS hospital bed stock reduced by 8.3% between 2010/2011 and 2019/20 as the average daily total of available beds fell from 153,725 to 140,978 (in 1987/88 there were 299,000 beds). Issues around bed occupancy are compounded by discharge delays caused by pressures in social care. Social care has been neglected by successive political regimes and remains on the backburner despite multiple political promises to the contrary. The UK in general continues to have a very low total number of hospital beds relative to its population: the average number of beds per 1,000 people in both OECD and EU countries is 5, while the UK has just 2.4 (Germany has 7.8).
- These general data on NHS expenditure and hospital capacity and care accessibility have clear implications for general practice, the core topic here. GP appointment bookings peaked over the winter of 2021. In terms of access, 48.1% of appointments in December 2022 were booked to take place on the same day (85% were booked to take place within two weeks); in terms of ‘appointment mode’, 68.3% of appointments were booked to take place face-to-face. At the same time, a number of practices have closed and more than two in five (42%) GPs are planning to work more flexibly and from home more. A long-term decline in GPs coincides with a rise in patient numbers. While there are 1,990 fewer fully qualified “full-time-equivalent” (FTE) GPs now than there were in September 2015, each practice has on average 2,224 more patients than in 2015. The average number of patients each GP is responsible for has increased by 335–17% – since 2015, and now stands at 2,273. Since 2017 the number of GPs working full-time hours or more in GP practice-based settings has been steadily decreasing. At the same time the number of GPs choosing to work less than full-time has been climbing, probably because doctors are moving to working patterns that allow them better to control their hours and workloads to reduce stress, ill-health and burnout. In reality, however, many part-time GPs often work additional unpaid hours just to get through the number of appointments, essential patient follow-ups and administrative work. In December 2022 there were 36,622 fully qualified GPs working in the NHS in England; in FTE terms, this equates to 27,375 fully qualified GPs. The overall number of GPs has seen little growth since 2015 while the number of GP partners has declined significantly. In a BMA survey, one in 10 GPs said they planned to leave the NHS altogether after the pandemic. Government plans to reverse this problem have so far failed.

What this assemblage of data confirms is that while patient dissatisfaction with primary care is understandably high, this problem cannot simply be laid at the door of GPs behaviour. It is obviously linked, for example, to growing deficits in secondary care options and, especially, in the long-term collapse of social care. But we need to delve deeper yet. In what follows I draw on a previous detailed study of the sociology of health and health care (Scambler, 2018). I shall argue that the top-down NHS reorganisations post-2010 were indeed aimed at facilitating the involvement of the private sector, and that *the strategy adopted to accomplish this was to deliberately underfund the NHS to create sufficient public dissatisfaction to allow the government to call in for-profit providers to “come to the rescue of the NHS” without having to face a crisis of state legitimation* (Habermas, 1975). It is revealing in this connection that several Conservative MPs and donors hold paid positions and/or shares in private health care companies (see Scambler et al., 2021).

The decade of political austerity from 2010 to 2020 was the product of a pre-planned Conservative government strategy to push back on post-WW2 “welfare statist capitalism”; and the underfunding of the NHS was at the core of this strategy. It was a plan awaiting a propitious moment. Thatcherite Conservative MPs had come to see the NHS both as “socialism in practice” and as ripe for plunder by business. As welfare statist gave way to financialised or rentier capitalism, an emergent and ultimately ubiquitous neoliberal ideology afforded cover for this act of political sabotage. But political action typically has tap roots in social structure and culture. I have long argued that social class qua social structure remains a vital causal force in contemporary English society. I have referred elsewhere to a new, or revised, “class/command dynamic” in post-1970s rentier capitalism. “Big business” has always exerted its influence on government and on policy. But in rentier capitalism this influence has grown exponentially, giving a new bite to the formula: *capital buys power to make policy in its interests*. Stated more precisely, a tiny hard core of owners of capital (well under the 1% exposed by the Occupy Movement), comprising a global mix of financiers, major shareholders and CEOs, many of them transnational ‘nomads’ with no loyalty or commitment to the nation states in which they reside and operate, can now buy far more political power to influence policy in their interests than could their predecessors in welfare state capitalism. Arguably, they now have representatives in the Conservative cabinet, not least in the form of multimillionaire Prime Minister Rishi Sunak. In terms of the class/command dynamic, class relations have come to exercise more control over the command relations of the state. It is the reinvigorated class power that the less than 1% of ‘capital monopolists’ have come to enjoy in rentier capitalism that has enabled the Conservatives to set about undermining the NHS and opening it up for international profiteering. All the evidence from comparative studies tells us that introducing markets into the provision of health care is a regressive move, and as mentioned earlier it is a process already well underway.

My deliberately provocative “greedy bastards hypothesis” (GBH) was advanced to address not primarily the assault on the NHS but the reconfiguration of what have come to be called “social determinants of health”. *If you want to understand and explain poverty, study the wealthy*. The GBH asserts that the growing health inequalities documented in England since the 1980s, actually accentuated during the COVID pandemic (Marmot and Allen, 2020), also have their genesis in the strategic behaviours of the capital monopolists. It is their telling influence on policy via their attacks on the welfare state, the NHS, benefits, employment security, disability, pension entitlements, trade union rights, protests and so on that have amplified health threats to the poorest and most impoverished in society; and the evidence is entirely consonant with this thesis. Poorer lives have been disfigured and cut short during rentier capitalism in general and particularly during the years of austerity and since (Scambler, 2018, 2020).

The impact of the COVID pandemic had several sequelae of relevance to the analysis in this paper. First, it showed the deep and deepening fissures in our fractured society in even sharper relief; and it did so in the domain of people’s health via COVID-enhanced morbidity and mortality rates for those disadvantaged by structural relations of class and race in long-deprived regions, communities and neighbourhoods. Second, it shone a harsh spotlight on a decade of NHS underfunding and the resultant worsening of public access to over-stretched and under-staffed NHS services (see above). Third, it placed intolerable burdens on already overworked doctors, nurses, and allied health and community and residential care workers, resulting ultimately in high rates of job-stress, burnout and people leaving their jobs. Fourth, it illustrated just how readily a government can find a “magic money tree”, not only when it needs to bail out banks but when a crisis of state legitimacy is in the offing. Fifth, it provided efficient cover for a stealthy pursuit of the government’s longstanding agenda of involving for-profit companies in the provision of health care services in the name of “meeting the COVID challenge”. As COVID began to fade as an alarming threat, so too did the welfare-statist default option of the in-house provision of clinical services. And sixth, it exposed afresh the levels of political corruption in awarding contracts for ameliorating the effects of COVID to Conservative Party donors, network allies and friends (Scambler et al., 2021; Maugham, 2023). At the time of writing this, a post-COVID “cost of living crisis”, consequent on new Conservative Prime Minister Sunak’s political resurrection of economic austerity following the implosion of the short-lived, flailing Truss premiership, has not only led to widespread strikes, including but by no means confined to health workers in the guise of junior doctors, nurses and ambulance staff, but once again holds out the promise of an imminent crisis of legitimation.

### What is to be done?

I have elsewhere discussed the structural and cultural obstacles to the kind of social transformation that might allow for a lasting reinvigoration of institutions like the NHS, distinguishing in the process between “attainable” and “aspirational” reforms (Scambler, 2022). The objective here is more modest. It is to suggest ways in which the NHS might be “saved” in the absence of some sort of social revolution. It draws on the work of Murphy and Hines on behalf of Tax Research UK (2023). Their underlying premiss is that the NHS is currently underfunded by approximately £30 billion *per annum*, the culmination of ‘austerity in NHS funding since 2010’. In sum, there is a shortfall of more than £400 per person per year in NHS funding. The authors address various imaginative but orthodox options for addressing this shortfall:

- £10 billion of the funding could be raised by the additional taxes paid by those employed by the NHS to deliver the requisite services, were they to be “lured back to the service by better working conditions and higher pay” (many of them now work in lower-paid jobs in the private sector). The impact of the extra NHS spending on growth elsewhere in the economy is taken into account in this estimate.
- At least £5 billion could be raised from taxes paid by those able to return to the workforce, either because their own conditions will be sufficiently well managed to allow this or because those that they care for will enjoy better health, letting them return to work.

If half the funding required to return the NHS to a healthy functioning state could be generated from the benefits created by this additional spending, what about the other £15 billion?

- The government could simply opt to run a bigger deficit to satisfy the remaining £15 billion. The impact on national debt would be insignificant, at less than 0.6% of national debt (according to the criteria that “the government likes to state it *per annum*”).
- Alternatively, the Bank of England currently has in place “a quantitative tightening programme of selling the government debt that it owns that it bought under the quantitative easing programmes that paid for the banking crises of 2008/9, the Brexit crisis of 2016 and the Covid crisis of 2020/21”. If £15 billion of this programme was cancelled each year and bonds to fund the NHS were sold instead, this step would deliver the necessary NHS funding. In such a case there would be no net impact on the amount of national debt owned by third parties.

Monies might also be raised via changes to the tax system that would have no effect on the vast majority of taxpayers:

- The tax reliefs on savings available to the wealthiest 10% of citizens each year might be halved. Presently this group enjoys at least £30 billion of pension and ISA tax reliefs each year. That subsidy per wealthy person might exceed average Universal Credit payments to each person in receipt of that benefit. Halving this relief would still provide the wealthy with very generous subsidies for their savings but would underwrite the NHS “we all need”.
- Or, given that the Public Accounts Committee of the House of Commons has found that for every £1 spent on tax investigations £18 of additional tax is raised, investing £1 billion in additional funding with HM Revenue and Customs might be sufficient to recover the funds required for the NHS each year.
- Another option might be to set the rate of capital gains tax, currently set at half the rate of income tax in most cases, and very largely paid by the wealthiest groups in society, at the same rate as the income tax rate. The revenue from this tax might then double, raising £15 billion per year.
- Other options might include: (i) raising £6 billion a year by charging an additional 15% income tax on the investment income of those below pensionable age who have more than £5,000 of investment income *per annum* (since they do not pay national insurance but enjoy the benefits of the NHS); or (ii) the so-called “non-dom” rule that lets wealthy people with an origin outside the UK live here but not pay tax on their overseas income could be abolished, raising in the region of £3 billion per year.

I am not economist, but it is clear from this “attainable” menu of options that there exist several routes out of the NHS underfunding that do not require the overthrow of rentier capitalism. That there is no prospect of any of them being followed at the time of writing bears testimony to the Conservative’s commitment to destroying rather than saving the NHS and, at a deeper or more sociological level, to the salience of the class/command dynamic.

### Concluding comments

In this brief contribution a personal narrative has been outlined as a way of introducing, preparing the ground for, and illustrating an analysis of the current state of play in the ailing NHS in England. It is not of course being claimed that this narrative is a substitute for a representative survey of the experiences of people-*cum*-patients, but we know enough from regular items on the mainstream and social media to suggest that it might well strike a chord. We also know that while patients are becoming increasingly dissatisfied with the health care on offer, they remain committed to the core principals of the NHS, namely, that it’s free at the point of use, available to everyone and funded by tax (Health Foundation poll, reported in an editorial in the Guardian, 2023). At the same time, the difficulty of accessing health care is prompting more people to jump NHS queues by “going private”. According to Dorling (2023), while in 1980 about 0.5% of Gross Domestic Product was spent on private health insurance, in 2021 it was more than 2%. In other words, *just as planned*, the involvement of the private sector in the provision of health care is growing apace. This brief paper has sought to place the day-to-day experiences of people-*cum*-patients like myself into a broader social and political context, and to link this to deeper structures like the changing relations between class and state. It is often GPs and GP receptionists who bear the brunt of people’s disillusionment.

My general assessment of a changing medical and health care environment might be summarised as follows. First, the medical and allied health professions should not only focus on extant problems with health care delivery but recognise and be upfront about the government’s agenda and direct responsibility for this deterioration. Second, the switch to phone calls and telemedicine as default options should be recognised for what it is, a coping device, and not presented as an innovation motivated by patient choice. This is *not* to reject a role for virtual expedients, far from it. But many third and fourth agers (like me) value *actual* contacts with *our* doctors, people who are familiar with us and our medical histories and have time enough to demonstrate through their attention and action that they *care* about our wellbeing. Finally, health care, just like population health, is intrinsically political. To pretend otherwise, or to remain institutionally or personally aloof, is effectively to genuflect to the status quo, a status quo which in contemporary England is irrational, exploitative and unacceptable. The “cost of living crisis”, and its articulation via the related “health care crisis”, might in the first half of 2023 yet issue in a crisis of state legitimacy and a more progressive than regressive package of reforms. We shall see.

## Data availability statement

The original contributions presented in the study are included in the article/supplementary material, further inquiries can be directed to the corresponding author.

## Author contributions

The author confirms being the sole contributor of this work and has approved it for publication.
